# Supplementary material for: Genomic epidemiology reveals the dominance of Hennepin County in the transmission of SARS-CoV-2 in Minnesota from 2020 to 2022
Source: mSphere. 2023 Oct 26;8(6):e00232-23. doi: 10.1128/msphere.00232-23 (PMC10871168; doi:10.1128/msphere.00232-23)
Supplement: Figure Captions — Captions for supplemental figures. [file msphere.00232-23-s0001.docx]

**SUPPLEMENTARY MATERIALS**

**FIGURE CAPTIONS**

**Fig. S1. Sequence distribution (n = 6,188) by Minnesota county/region by month for our phylodynamic analysis.** We re-created the legend and text labels in Adobe Illustrator for visualization purposes.

**Fig. S2. Supported routes of pairwise SARs-CoV-2 transmission as determined by the Bayes factor (BF) statistic.** Here, we show routes with a BF > 100. The darker the lines, the higher the BF (grey to black).

**Fig. 3. Phylogeny of 24,070 full genome SARS-CoV-2 sequences generated for this study from 2020-2022 via Nextstrain (augur v15.0.2).** Branch colors indicate the assigned clade.

**Fig. S4. Map of Minnesota counties included in the phylodynamic analysis.** Counties with the same color are part of a discrete region (northern, central, and southern) used in the analysis. We show an inset of a portion of the map for visualization purposes.

**Fig. S5. Virus diversity and cases per county/location.** On the top of each panel, we show the normalized Shannon diversity index over time for each Minnesota county or region in the study. The shaded areas represent the 95% Bayesian highest posterior density (HPD). Below, we show the seven-day average cases for the particular county or region obtained from [1] via outbreak.info. We aggregated the data for Northern, Central, and Southern Minnesota based on the counties listed in Table S1. The first vertical line indicates the end of lockdown in Minnesota on May 18, 2020 [2]. The second vertical line indicates the end of all COVID-19 restrictions in the State on May 28, 2021 [3]. We combined location-specific graphs and re-created the location labels in Adobe Illustrator for visualization purposes.

**REFERENCES**

1. Dong, E., H. Du, and L. Gardner, *An interactive web-based dashboard to track COVID-19 in real time.* Lancet Infect Dis, 2020. **20**(5): p. 533-534.

2. *Governor Walz Extends Stay Home Order in Minnesota*. 2020 Apr 30; Available from: <https://mn.gov/governor/news/?id=1055-430501>.

3. *Impact of Opening and Closing Decisions by state*. 2022 [cited 2022 Apr 28]; Available from: <https://coronavirus.jhu.edu/data/state-timeline/new-confirmed-cases/minnesota/99>.
