# Supplementary figures and images for: Genomic epidemiology reveals the dominance of Hennepin County in the transmission of SARS-CoV-2 in Minnesota from 2020 to 2022
Source: mSphere. 2023 Oct 26;8(6):e00232-23. doi: 10.1128/msphere.00232-23 (PMC10871168; doi:10.1128/msphere.00232-23)

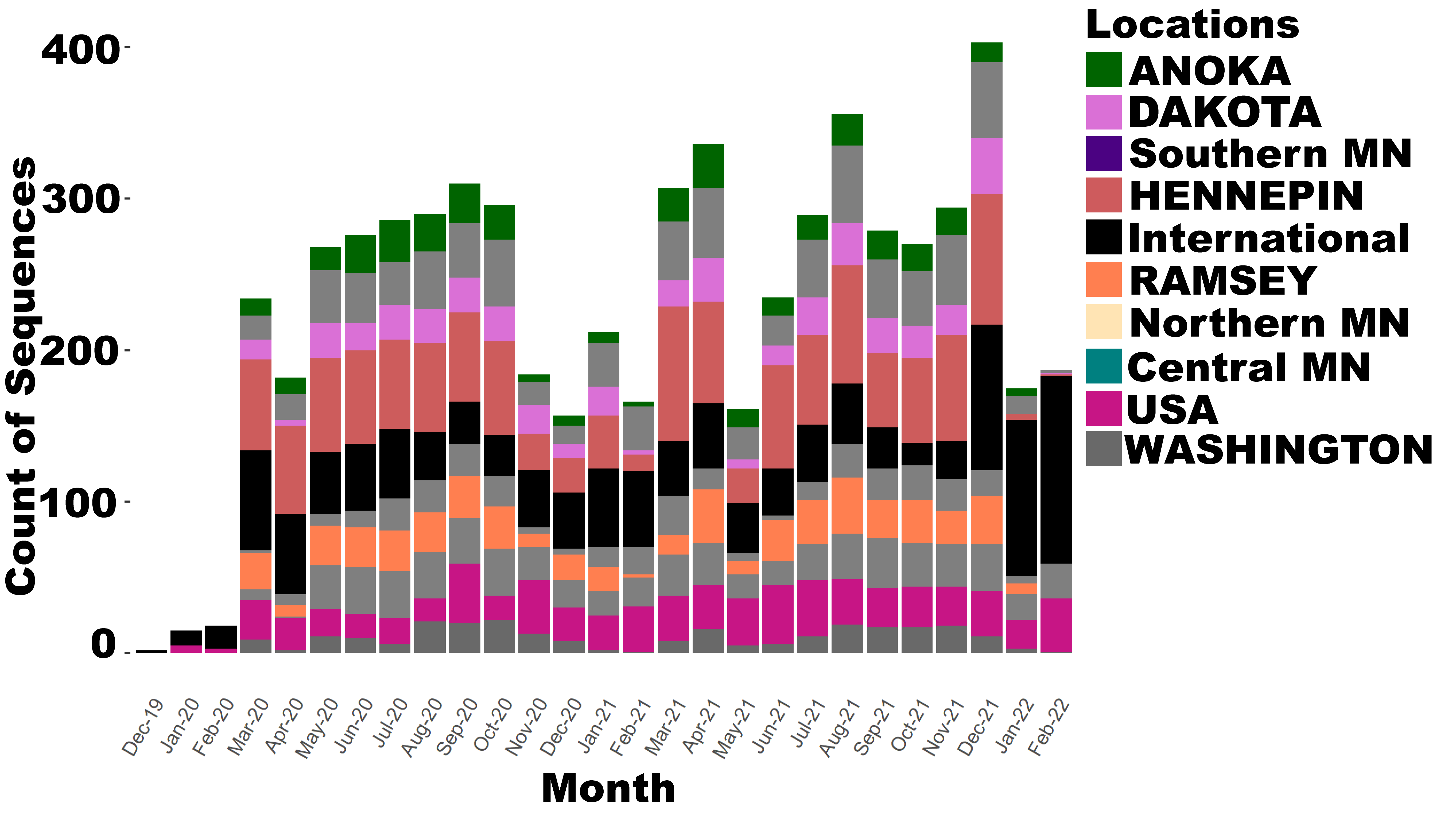

Supplement: Fig. S1 — Sequence distribution (n = 6,188) by Minnesota county/region by month for our phylodynamic analysis. [file msphere.00232-23-s0002.tif]

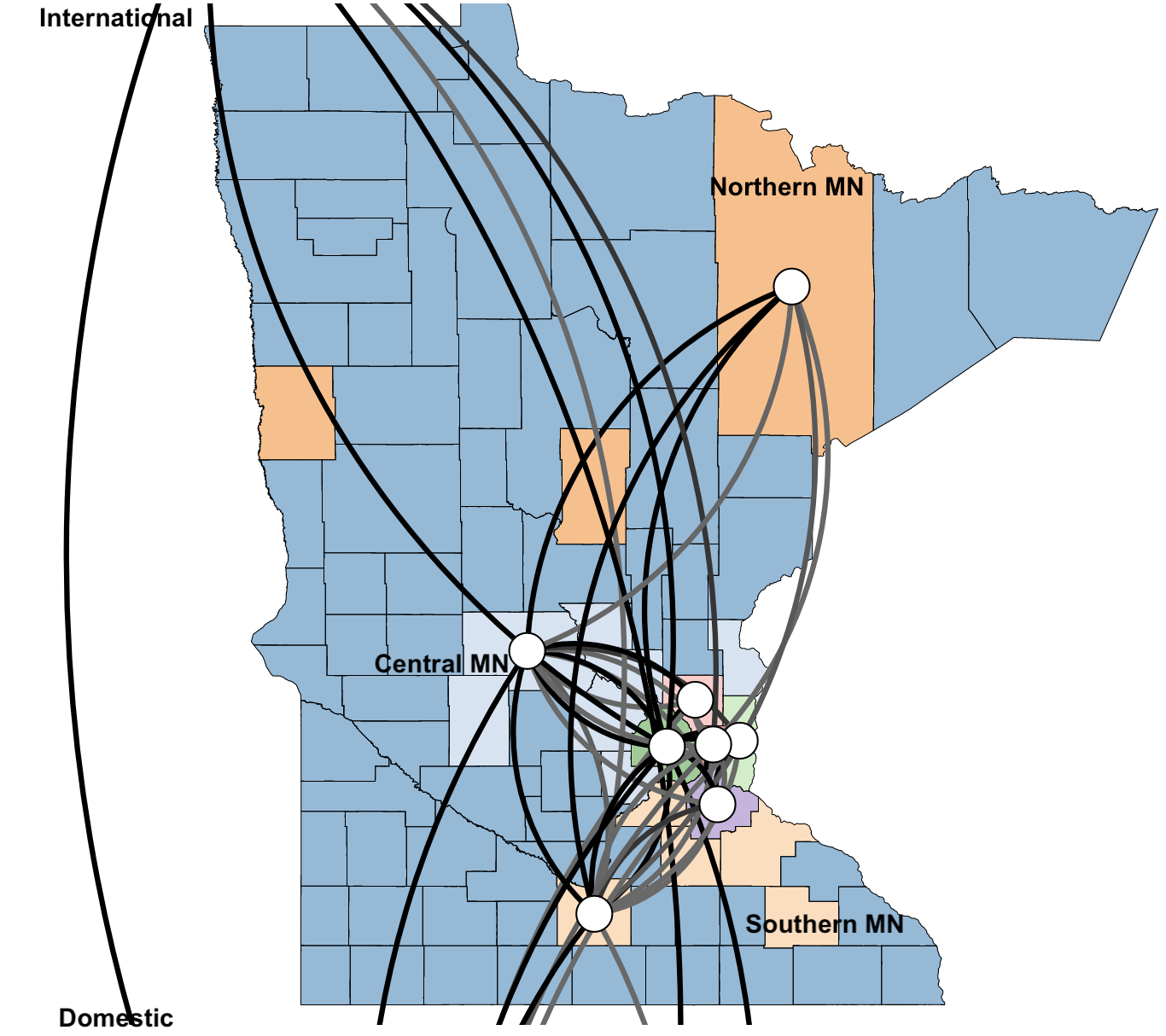

Supplement: Fig. S2 — Supported routes of pairwise SARs-CoV-2 transmission as determined by the Bayes factor (BF) statistic. [file msphere.00232-23-s0003.tif]

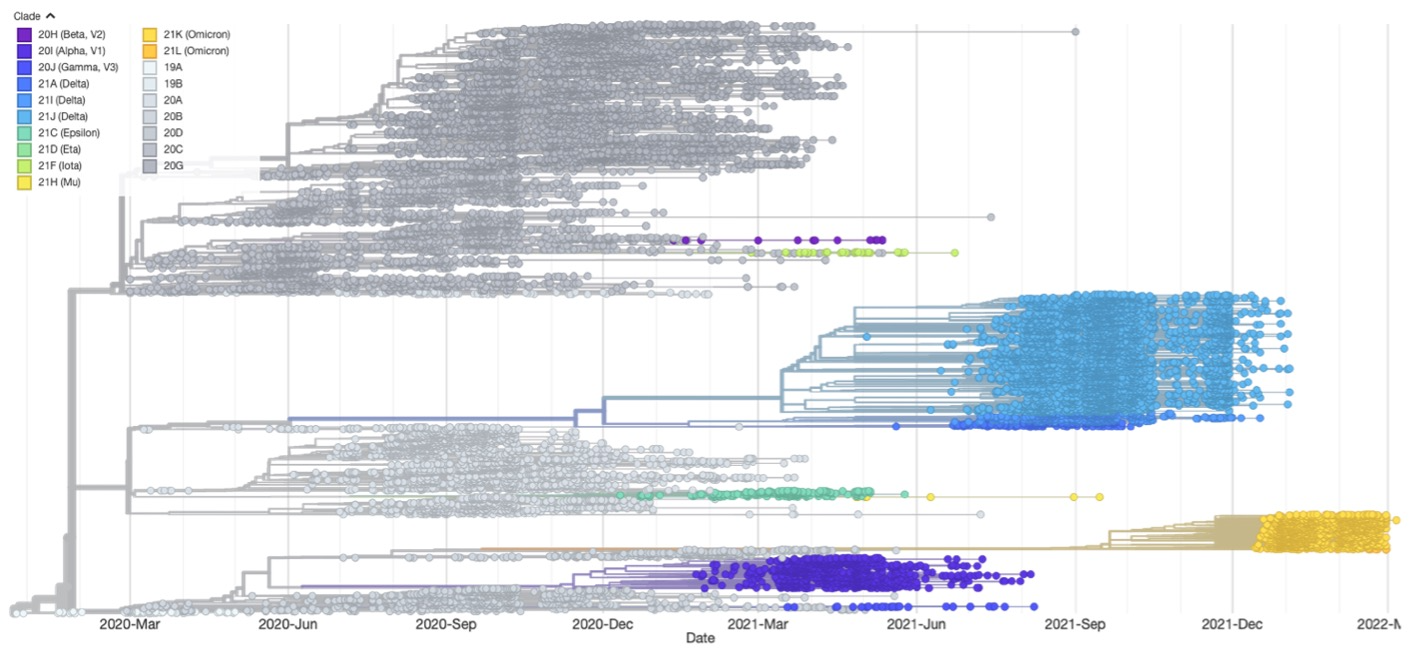

Supplement: Fig. S3 — Phylogeny of 24,070 full genome SARS-CoV-2 sequences generated for this study from 2020-2022 via Nextstrain (augur v15.0.2). [file msphere.00232-23-s0004.tif]

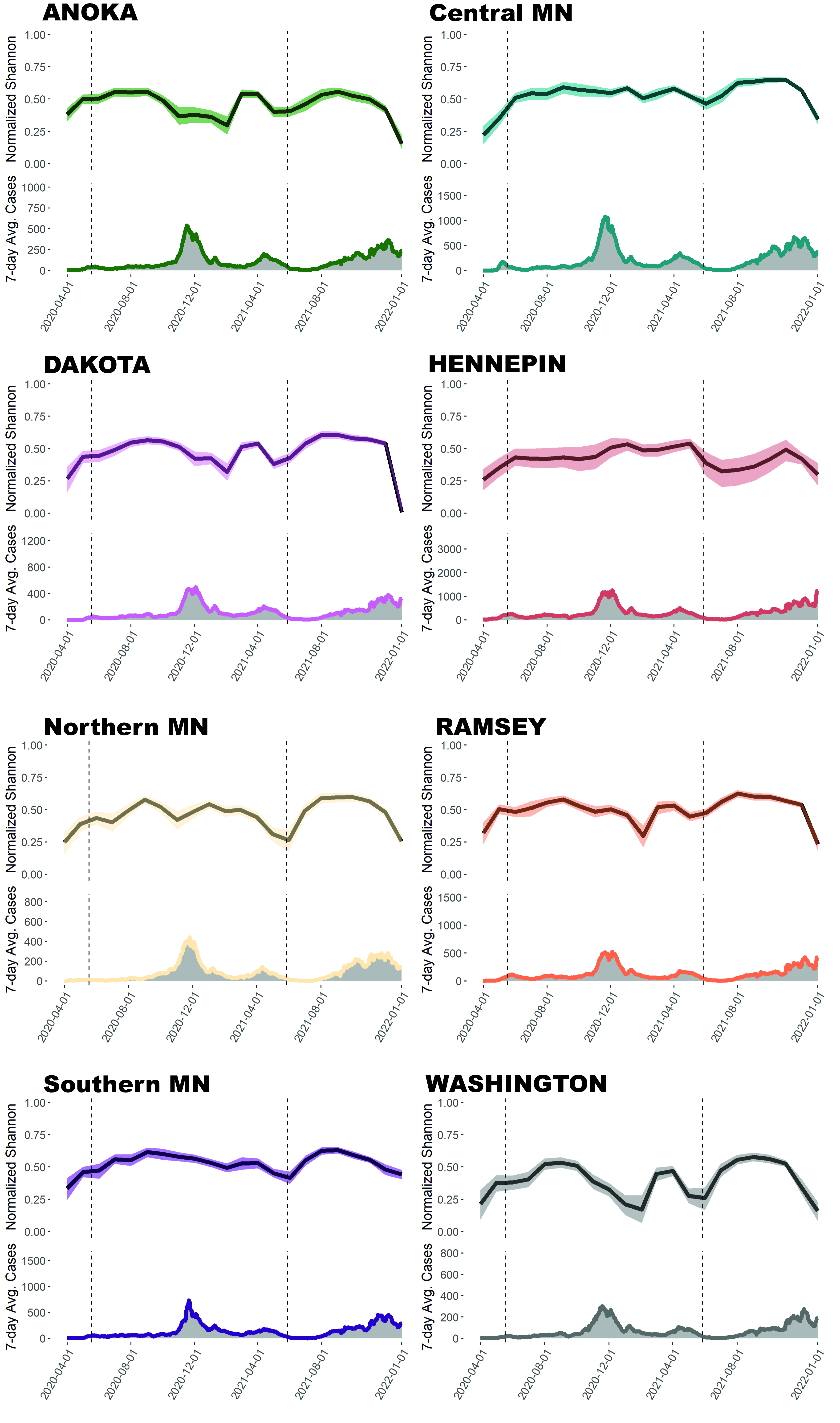

Supplement: Fig. S5 — Virus diversity and cases per county/location. [file msphere.00232-23-s0006.tif]
