# Supplementary material for: Genomic epidemiology reveals the dominance of Hennepin County in the transmission of SARS-CoV-2 in Minnesota from 2020 to 2022
Source: mSphere. 2023 Oct 26;8(6):e00232-23. doi: 10.1128/msphere.00232-23 (PMC10871168; doi:10.1128/msphere.00232-23)
Supplement: Supplemental Tables — Tables S1 to S4. [file msphere.00232-23-s0007.docx]

**SUPPLEMENTARY MATERIALS**

**TABLES**

**Table S1. Number of sequences by location for the phylodynamic analysis.**

| **Location** | **Number of Sequences (n = 6,188)** |
| --- | --- |
| Anoka | 371 |
| Dakota | 419 |
| Hennepin | 1,224 |
| Ramsey | 501 |
| Washington | 257 |
| Central Minnesota* | 732 |
| Northern Minnesota^$^ | 319 |
| Southern Minnesota^#^ | 568 |
| International | 1,152 |
| USA | 645 |

^*^Central Minnesota includes seven counties: Benton, Carver, Chisago, Kandiyohi, Sherburne, Stearns, and Wright. ^$^Northern Minnesota includes three counties: Clay, Crow Wing, and Saint Louis. ^#^Southern Minnesota includes five counties: Blue Earth, Goodhue, Olmsted, Rice, and Scott.

**Table S2. Log marginal likelihood values calculated via stepping-stone and path-sampling for different coalescent priors under a strict molecular clock.** The results favor the use of a non-parametric Skygrid coalescent model.

| **Tree Prior** | **Stepping-Stone** | **Path-Sampling** |
| --- | --- | --- |
| Skygrid | -12,690.78 | -12,683.44 |
| Constant | -13,041.41 | -13,033.6 |

**Table S3. Bayes factor and posterior probability values for support of non-zero SARS-CoV-2 transmission of Minnesota counties that met our *a priori* threshold > 100.** We highlight the 48 routes out of 90 in this category in grey. We omitted routes that did not involve Minnesota such as USA🡪International or International 🡪USA. Hennepin was most often included as the origin (9/48). Meanwhile, Southern MN was most popular as the destination (8/48), followed by Ramsey (7/48) and Central MN (7/48).

| **From** | **To** | **Bayes Factor** | **Posterior Probability** |
| --- | --- | --- | --- |
| Central MN | HENNEPIN | 74,564.19 | 1 |
| Central MN | Northern MN | 74,564.19 | 1 |
| DAKOTA | RAMSEY | 74,564.19 | 1 |
| DAKOTA | WASHINGTON | 74,564.19 | 1 |
| HENNEPIN | International | 74,564.19 | 1 |
| HENNEPIN | Northern MN | 74,564.19 | 1 |
| HENNEPIN | RAMSEY | 74,564.19 | 1 |
| HENNEPIN | Southern MN | 74,564.19 | 1 |
| HENNEPIN | USA | 74,564.19 | 1 |
| HENNEPIN | WASHINGTON | 74,564.19 | 1 |
| International | USA | 74,564.19 | 1 |
| RAMSEY | WASHINGTON | 74,564.19 | 1 |
| Central MN | ANOKA | 74,564.19 | 1 |
| HENNEPIN | ANOKA | 74,564.19 | 1 |
| HENNEPIN | Central MN | 74,564.19 | 1 |
| Southern MN | Central MN | 74,564.19 | 1 |
| USA | Central MN | 74,564.19 | 1 |
| HENNEPIN | DAKOTA | 74,564.19 | 1 |
| International | HENNEPIN | 74,564.19 | 1 |
| Southern MN | HENNEPIN | 74,564.19 | 1 |
| USA | HENNEPIN | 74,564.19 | 1 |
| USA | International | 74,564.19 | 1 |
| Southern MN | Northern MN | 74,564.19 | 1 |
| USA | Southern MN | 74,564.19 | 1 |
| Central MN | WASHINGTON | 37,277.95 | 1 |
| International | RAMSEY | 37,277.95 | 1 |
| Southern MN | DAKOTA | 37,277.95 | 1 |
| Northern MN | Southern MN | 12,420.46 | 0.999 |
| Central MN | Southern MN | 7,448.96 | 0.999 |
| USA | RAMSEY | 6,771.03 | 0.999 |
| RAMSEY | DAKOTA | 4,134.63 | 0.998 |
| Northern MN | Central MN | 2,477.46 | 0.997 |
| Southern MN | RAMSEY | 2,251.49 | 0.996 |
| Central MN | RAMSEY | 2,063.17 | 0.996 |
| ANOKA | Central MN | 1,954.15 | 0.996 |
| Northern MN | RAMSEY | 1,214.21 | 0.993 |
| RAMSEY | Central MN | 999.45 | 0.992 |
| Central MN | DAKOTA | 460.72 | 0.982 |
| Southern MN | USA | 403.72 | 0.98 |
| USA | ANOKA | 320.23 | 0.975 |
| RAMSEY | ANOKA | 285.31 | 0.972 |
| RAMSEY | HENNEPIN | 276.34 | 0.971 |
| RAMSEY | Southern MN | 266.89 | 0.97 |
| DAKOTA | Central MN | 229.21 | 0.965 |
| DAKOTA | Southern MN | 213.66 | 0.963 |
| WASHINGTON | Southern MN | 187.44 | 0.958 |
| USA | WASHINGTON | 148.05 | 0.947 |
| International | Southern MN | 136.24 | 0.943 |
| International | Central MN | 95.72 | 0.92 |
| USA | Northern MN | 58.84 | 0.877 |
| Southern MN | International | 51.76 | 0.862 |
| Southern MN | ANOKA | 42.38 | 0.836 |
| DAKOTA | Northern MN | 32.64 | 0.798 |
| Northern MN | DAKOTA | 30.72 | 0.788 |
| International | ANOKA | 30.1 | 0.784 |
| International | WASHINGTON | 20.69 | 0.714 |
| ANOKA | RAMSEY | 16.33 | 0.663 |
| Northern MN | HENNEPIN | 14.37 | 0.634 |
| WASHINGTON | ANOKA | 12.64 | 0.604 |
| ANOKA | HENNEPIN | 7.95 | 0.49 |
| Southern MN | WASHINGTON | 3.65 | 0.306 |
| Central MN | USA | 3.48 | 0.296 |
| DAKOTA | HENNEPIN | 3.28 | 0.283 |
| ANOKA | DAKOTA | 2.47 | 0.23 |
| DAKOTA | ANOKA | 2.4 | 0.225 |
| ANOKA | WASHINGTON | 2.24 | 0.213 |
| RAMSEY | Northern MN | 2.13 | 0.205 |
| ANOKA | USA | 1.9 | 0.186 |
| ANOKA | Southern MN | 1.49 | 0.153 |
| Northern MN | USA | 1.47 | 0.151 |
| Northern MN | WASHINGTON | 1.3 | 0.135 |
| WASHINGTON | RAMSEY | 1.27 | 0.133 |
| WASHINGTON | DAKOTA | 1.17 | 0.123 |
| WASHINGTON | HENNEPIN | 1.15 | 0.121 |
| RAMSEY | USA | 1.1 | 0.117 |
| WASHINGTON | Central MN | 0.93 | 0.101 |
| DAKOTA | USA | 0.83 | 0.091 |
| WASHINGTON | Northern MN | 0.72 | 0.08 |
| International | DAKOTA | 0.68 | 0.076 |
| Northern MN | International | 0.64 | 0.072 |
| Central MN | International | 0.43 | 0.049 |
| USA | DAKOTA | 0.4 | 0.047 |
| RAMSEY | International | 0.28 | 0.033 |
| International | Northern MN | 0.28 | 0.032 |
| WASHINGTON | International | 0.25 | 0.03 |
| WASHINGTON | USA | 0.24 | 0.028 |
| ANOKA | Northern MN | 0.17 | 0.02 |
| DAKOTA | International | 0.15 | 0.017 |
| ANOKA | International | 0.13 | 0.016 |
| Northern MN | ANOKA | 0.11 | 0.013 |

**Table S4. Demographics of N = 22,514 Mayo Clinic Laboratories COVID-19 patients included in the study.**

| **Demographic** | **Category** | **Count (%)** |
| --- | --- | --- |
| Gender | Male | 11,423 (49) |
|  | Female | 11,076 (51) |
|  | Non-binary | 15 (<1) |
|  |  |  |
| Age | < 18 | 3,416 (15) |
|  | 18-45 | 11,204 (50) |
|  | 46-64 | 5,350 (24) |
|  | 65+ | 2,544 (11) |
|  |  |  |
| State |  |  |
|  | Minnesota | 21,669 (96) |
|  | Wisconsin | 413 (2) |
|  | Iowa | 184 (1) |
|  | Other States | 148 (1) |
